# Voters’ short-term responsiveness to coalition deals

**DOI:** 10.1177/13540688211029794

**Published:** 2021-07-05

**Authors:** Carolina Plescia

**Affiliations:** University of Vienna, Austria

**Keywords:** coalition deals, government formation, voters’ perceptions

## Abstract

Government formation in multiparty systems requires election winners to strike deals to form a coalition government. Do voters respond and how do they respond to coalition government deals? This paper examines the short-term consequences of coalition government formation on voters in European democracies relying on survey panel data and original content analysis of coalition agreements. It tests theoretical expectations that deal with both the actual and perceived ideological shifts parties make when joining coalition deals as well as the effect of a much simpler heuristic cue based on preferences. The findings indicate that coalition deals have consequences on party preferences, but voter perceptions play a much stronger effect than the actual content of coalition deals. These results have important implications for our understanding of public opinion and provide important insights into the current difficulties and challenges of government formation and representative democracy.

## Introduction

There is no shortage of research on what Mayhew (1974) has canonically defined as the mass-party ‘electoral connection’. Given that citizens are primarily represented by and through parties, it is often considered normatively desirable that parties’ policy positions match the views of their supporters and that voters respond by updating their perceptions when parties’ positions change (Downs, 1957). As of today, there is more evidence that political parties respond to shifts in voter preferences (e.g., Adams et al., 2006, 2009) and listen to voter issue priorities (e.g., Klüver and Spoon, 2014; Spoon and Klüver, 2014) than evidence that voters actually perceive parties’ policy shifts, and that these shifts have significant electoral consequences (but see Adams et al., 2011; Fernandez-Vazquez, 2014; Tavits, 2007). There is, however, evidence that voters respond to parties’ observable actions while in government both in the US (e.g., Ansolabehere and Jones, 2010; Levendusky, 2009) and in European contexts with coalition governments (e.g., Fortunato, 2017; Fortunato and Stevenson, 2013; Matthieß, 2020).

The literature to date, however, has not examined voters’ responsiveness to the deals parties have to make right after the election to form a government. In parliamentary democracies, elections commonly require the ‘winners’ of the elections to compromise to form a coalition government; in forming such governments parties may need to shift ideologically and the coalition deal will eventually determine the ideological orientation of the next government (Strøm and Müller, 1999).^1^ As Strøm (2008) explained, the ultimate mass-elite ‘electoral connection’ in legislative elections in multiparty systems is via such government formation. It is thus important to assess whether voters remain oblivious to these coalition deals or whether they respond and with what consequences.

Building on different strands of existing research, this paper tests two main arguments. The first – and admittedly most demanding – argument expects voters to perceive the *actual* policy shifts parties make after the elections to join coalition governments, and that these shifts have significant consequences on party preferences (e.g., Adams et al., 2011; Fernandez-Vazquez, 2014; Tavits, 2007). The second argument is far less demanding and, in line with the work of Fortunato and Stevenson (2013), suggests that voters will more simply respond to the ‘action’ of coalition government formation without taking the actual ideological movements as detailed in the government deal into account. In this latter case, voters’ response to parties’ policy shifts will be based on their perceptions of where parties now stand ideologically and/or on coalition preferences.

These two logics are tested combining survey panel data – interviewing the same respondents right before and after the election – with original manual content analysis of the coalition agreement signed by the coalition partners in eight elections in four countries namely Austria, Germany, Netherlands and United Kingdom.

The findings indicate that when the ideological distance between voters and parties increases after the election, there is a small but substantial decrease of party preferences compared to before the elections. I find, however, that voters mainly respond to the action of coalition government formation but hardly ‘notice’ how parties *actually* move.

The paper provides research findings on a neglected facet of coalition politics, that is, the voter-level ramifications of coalition agreements. In line with recent findings (Fernandez-Vazquez, 2014; Plescia and Staniek, 2017), this paper’s results challenge the view that voters are not responsive to parties’ policy shifts providing instead evidence that voters are more attentive than they appear. It shows that there is an almost immediate response to coalition deals which adds to our knowledge that coalition politics have long-term effects (Fortunato and Stevenson, 2013; Matthieß, 2020). This paper’s findings are also of interest for parties themselves because their electoral future depends on the extent to which voters accept or reject their coalition deals. In this paper, we learn that coalition agreements that move parties ideologically too far away from their voters are costly for parties themselves. While here I focus exclusively on the short-term perspective, it is clear that coalition formation is a dangerous strategy and it may be risky for parties to reveal their coalition preferences before the election. Understanding these patterns provides important insights into the difficulties and challenges of representative democracy, and the representation dilemma that political compromise poses for both parties and citizens as also discussed in the conclusion of this paper.

## Coalition politics and voters: The story thus far

There is an extensive literature on coalition politics about the making and breaking of governments (Laver and Schofield, 1998) as well as on coalition management (e.g., Müller and Strøm, 2000), its determinants (e.g., Martin and Vanberg, 2011) and its policy consequences (Miller and Müller, 2010). Yet, until very recently, we knew virtually nothing about voter reaction to coalition government politics.

In recent years, political science research has increasingly directed its attention towards coalition government politics as an integral part of the decision-making calculus of voters. Current research has been developing in two main directions.

On the one hand, existing studies have focused on the consequences that being part of a coalition government has on parties’ perceived policy positions after a full term in office. Bawn and Somer-Topcu (2012), for instance, found that voters tend to ‘discount’ the policy pronouncements of members of the incumbent coalition during the election campaign. Similarly, a series of recent studies has shown that voters perceive the positions of parties that have shared power in coalition governments as being more similar (e.g., Fortunato and Stevenson, 2013). Relatedly, a study by Spoon and Klüver (2017) indicates that conflict between coalition partners can reduce voters’ misperception of coalition parties’ policy positions. A recent work by Fortunato (2017) even indicates that the compromise parties have to make while in government is detrimental to their reputation and likely to be punished by voters in the next election.

On the other hand, the existing literature has demonstrated that voters mind which type of government will form after the election (Kedar, 2005) and in multiparty systems, voters consider not only the programmatic offer of parties but also coalition formation processes and coalition bargaining when casting their vote (Duch et al., 2010; Meffert and Gschwend, 2010).

These findings are significant because they imply that voters are aware of coalition politics and this has important consequences on their perceptions of parties, political behaviour and partisan preferences. Yet, there has been very little research investigating *whether* and *how* voters react to the deals parties are normally required to make right after the election in order to form coalition governments.^2^ The lack of research investigating voter reactions to coalition formation is puzzling considering not only how important these agreements are in terms of (effective) policymaking (e.g., Bäck et al., 2017; Strøm et al., 2008) and voting behaviour during election times (Matthieß, 2020), but also because of the media and political attention they attract in the aftermath of the elections. This paper takes up the task of filling in this research gap by investigating *whether* and *how* voters react to coalition government deals immediately after the elections.

## Voters’ reactions to coalition agreements in the aftermath of the election: Hypotheses

The focus of this paper is on voters’ reaction to coalition government formation. The obvious starting point of the theory is Downs’ (1957) classic idea that voters choose one party over the others based on the relative ideological distance between them and all the parties. As the party’s position further deviates from a voter’s ideal position, the voter receives less utility from voting for that party. The parties involved in coalition agreements usually differ in terms of the policy positions they had during the election campaign. The coalition agreements they sign after the election represent an (ideological) bargaining result among at least two parties; the existing literature on coalition politics has shown that parties vary widely in their ability to reach agreements that represent their ‘own’ policy preferences (e.g. Bäck et al., 2011; Schermann and Ennser-Jedenastik, 2014). And often coalition partners must make some fundamental programmatic shifts if the coalition is to be viable and at least somewhat effective in governance (Banaszak and Doerschler, 2012).

The existing literature also seems to suggest that voters do understand parties’ necessity to compromise to form a government (see Kedar, 2005). For example, when examining voters’ preferences *before* the elections, Gschwend and Hooghe (2008) and Plescia (2017) find that voters are less likely to prefer coalitions if they expect too many policy concessions to be made when in government. Similarly, after the election, the coalition politics literature shows that, to avoid as much as possible drafting agreements that their supporters may not like, parties seek coalition partners that are closest to their ideal position (e.g., Strøm et al., 2010).^3^

When two or more parties reach a coalition agreement, they depolarise ideologically and attenuate their overall stances towards those whom they had opposed during election campaigns (Curini and Hino, 2012). This ideological depolarisation implies ideological movements that can bring parties further away from or closer to the position of voters. When parties move away from voters, the lower utility that the increased distance implies for voters should be reflected in a decrease in party preferences. This argument leads to the first hypothesis:

**Hypothesis 1:** An increase (decrease) in the ideological distance between the voter and the party after the election leads to a decrease (increase) in party preferences.

Hypothesis 1 assumes that voters know which parties have formed a coalition government and are aware of parties’ ideological movements to form such a coalition. While it is unlikely that the typical voter will read the coalition agreement line by line, there are several reasons why the assumption behind Hypothesis 1 might still hold true. First, coalition formation receives large attention from the media (Costello and Thomson, 2007). This media attention seems to have even increased over time as striking coalition deals seems to have become more difficult for parties (Ecker and Meyer, 2017). Second, and relatedly, average levels of political knowledge are usually the highest immediately following election campaigns (Andersen et al., 2005).

Notwithstanding, sceptics may contend that Hypothesis 1 is too demanding for the typical voter. After all, there is almost near consensus (albeit relatively little empirical evidence outside the US) that members of the public know little about politics (e.g., Converse, 1964). So, there are grounds to suspect that even if information about coalition deals and parties’ ideological movements may be relatively easy to come by, especially during election times, it can still be fairly difficult for a typical voter to apply the heuristic rule behind Hypothesis 1. Hence, one can put forward contending theoretical expectations that voters do not necessarily respond to the content of a coalition deal but more simply respond to a party’s ‘action’ of joining a government. In other words, and following Fortunato and Stevenson (2013) among others, I expect that coalition membership is the most accessible and only cue voters will use to update their preferences for parties immediately after the elections.

The first alternative hypothesis simplifies voters’ reasoning by expecting them to respond to parties’ ideological movements but as perceived by voters, not necessarily the actual movements parties make to form the coalition agreement. This implies that voters will not respond to parties’ actual movements but to the movements voters perceive they have made. Over the years, the existing literature has shown that voters have an understanding of where parties stand ideologically (e.g., Dalton et al., 2011; Fernandez-Vazquez, 2014) albeit these perceptions do not necessarily always correspond to the actual positions of parties as measured for example by parties’ election manifestos (e.g., Adams et al., 2011, 2016; Spoon and Klüver, 2017). This leads to the next hypothesis:

**Hypothesis 2:** An increase (decrease) in the *perceived* ideological distance between the voter and the party after the election leads to a decrease (increase) in party preferences.

A second and final alternative hypothesis is that voters do respond to coalition deals but without considering how much or where parties have moved ideologically to join that coalition government. Again, coalition membership is the most accessible and only cue voters will use to update their preferences for parties after the elections, but this time I do not even expect them to reason ‘ideologically’. Simply put, voters will respond positively to coalition governments they like and negatively to those that include parties they dislike regardless of the ideological shifts parties make to join coalition governments. This leads to the last hypothesis:

**Hypothesis 3:** An increase (decrease) in coalition preferences leads to an increase (decrease) in party preferences.

## Data

To test the hypotheses of this study, I rely on existing national election panel studies that include a pre- and post-election *panel* component and include, for the same respondents and in both waves, questions on party preferences. The countries for which these data are available are Austria, Germany, the Netherlands and United Kingdom. Using this survey data, I calculate for each respondent the difference between party preferences in the survey wave immediately before the election and after the election and this constitutes the dependent variable of this study.

**Figure 1.**
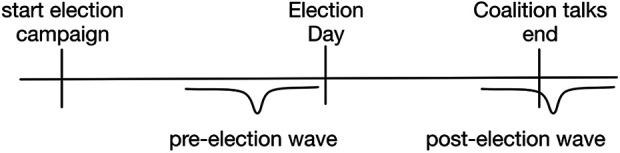
Timeline of survey data collection.

Figure 1 shows schematically the logic of the timing of the dependent variable: the pre-election data are collected during the week immediately before the election; in the aftermaths of the election, data collection for the post-election wave starts. The post-election wave data collection overlaps with coalition formation. As shown in Figure 1, some respondents are interviewed right after the coalition deal has been signed but in some cases respondents might have been interviewed just a few days before coalition talks have formally ended.

Instead of excluding the respondents interviewed before the signing of the coalition deal, I created a sample weight aimed at weighting each respondent on the basis of the timing of their post-election interview. Those that were interviewed after the coalition deal had been formally signed received a weight of 100% (regardless of the actual day of the interview); those that had been interviewed before received a weight that decreased as one moved back in time from the end of the coalition talks and closer to the Election Day.^4^ Since the timing of the interview is not random, retaining only the respondents that have been interviewed after the completion of the coalition talks brings the risk of using an unrepresentative sample of respondents. In addition, while the formal signing of the coalition deal does indeed mark a pivotal moment in coalition talks, discussions before that pivotal moment are almost equally important in terms of voters’ knowledge, especially in the few days immediately prior to the signing of the coalition agreement by the parties. Hence, the decision to retain the full sample of respondents interviewed after the elections.^5^

Table 1 provides an overview of the case studies included in the analysis. For Austria 2017 and Germany 2017, the full sample of respondents was interviewed after the coalition agreement was signed. In Germany 2009, this percentage is about 41%, in Germany 2013 it is 32% and about 97% in the United Kingdom. In the Netherlands in 2006, none of the respondents was interviewed after the coalition agreement was signed but about half of the sample was interviewed just a few weeks before coalition talks ended for the formation of the Fourth Balkenende cabinet. Importantly, the analysed countries differ quite substantially in terms of coalition agreements and government formation. Specifically, Austria is a case of grand-coalition governments with relatively short coalition talks. The elections included for Germany span grand-coalition governments and other coalitions, again with relatively short rounds of coalition talks with the exception of 2017. The Fourth Balkenende cabinet in the Netherlands in 2007 followed a turbulent time in Dutch politics and was the result of prolonged negotiations among the many winners and losers of the 2006 elections. The United Kingdom represents a case of coalition formation under a majoritarian system, which led to a coalition government in 2010 between the Conservative and the Liberal Democratic party. This variation is important and provides additional ground to test the robustness of the empirical findings of this paper. The country selection covers not only grand coalitions and coalitions with a larger and smaller coalition partner but also countries with a long history of coalition governments as well as countries such as the UK where coalitions are rather an unusual phenomenon

**Table 1.** List of country-elections included in the study.

**COUNTRY**	**ELECTION DATE**	**COALITION TALKS**	**PARTIES**
Austria	15.10.2017	began: 25.10.2017; ended: 15.12.2017	ÖVP-FPÖ
Austria	29.09.2013	began: 16.11.2013; ended: 13.12.2013	SPÖ-ÖVP
Germany	24.09.2017	began: 21.01.18; ended: 07.02.2018 (signed on 12.03.2018)	CDU-SPD
Germany	22.09.2013	began: 23.10.2013; ended: 27.11.2013 (signed on 14.12.2013)	CDU-SPD
Germany	27.09.2009	began: 05.10.09; ended: 24.10.09	CDU-FDP
Germany	18.09.2005	began: 17.10.05; ended: 11.11.05	CDU-SPD
Netherlands	22.11.2006	second round: 20.12.2006 to 22.02.2007	CDA-PvdA-Christian Union
United Kingdom	06.05.2010	began: 07.05.2010; ended: 12.05.2010	Conservative-Liberals

*Notes*: The surveys were administered by the Austrian National Election Study (Kritzinger et al., 2014; Wagner et al., 2018); the German National Election Study 2017 (Roßteutscher et al., 2018); the Short-term Campaign Panel for 2009 and 2013 (Rattinger et al., 2015); the Campaign panel for 2005 (Schmitt-Beck and Faas, 2009); the Dutch Parliamentary Election Study (van der Kolk et al., 2006) and the British Election Study 2009–2010, respectively. The survey waves used in this paper are wave 4 and wave 6 for Austria 2017; wave 1 and wave 2 for Austria 2013; wave 7 and wave 9 for Germany 2017; Vorwahlwelle and Nachwahlwelle for Germany 2013; wave 6 and wave 7 for Germany 2009; Vorwahlwelle and Nachwahlwelle for Germany 2005; wave 2 and wave 3 for the Netherlands and the pre- and post-election wave for the UK.

## Variables and models

The unit of analysis is each survey respondent. The dependent variable is, for each respondent, the difference between the party preferences respectively before and after the election. Since party preferences are measured using a scale from 0 to 10 where ‘0’ means ‘do not like the party at all’ and ‘10’ means ‘like the party very much’, the dependent variable can theoretically range from −10 to +10 with increasing values representing an increase of party preferences after the election. I will run separate empirical models respectively for the prime minister’s party and its junior coalition partner.^6^

The first key independent variable is the *difference* between two absolute differences: one between the ideological position of the voter ( $V_{before}$ ) and that of the coalition agreement ( $C_{after}$ ) after the election, and the second between the ideological position of the voter ( $V_{before}$ ) and that of the party signing the agreement ( $P_{before}$ ) before the election, that is:


$$change\;in\;actual\;distance=\left|V_{before}-C_{after}\right|-\left|V_{before}-P_{before}\right|$$

Assuming the ideological position of voters did not change after the election,^7^ increasing values of this independent variable represent an increase in the distance voter-party after the election compared to before the election. I use this variable to test *Hypothesis 1*.

The ideological position of the voter ( $V_{before})$ is taken directly from the national election surveys used in this paper and is measured using an 11-point scale from 0 = left to 10 = right. Given that not all the survey data used in this paper contain information on voters’ ideological position on specific policy issues like immigration, economy or the environment, it is unfortunately not possible to test the theoretical expectations for any specific policy issue. Still one has to consider that the overwhelming majority of the existing literature relies on a general left-right ideological scale since this still constitutes the primary dimension of conflict in most established democracies (Marks and Steenbergen, 2002).

While there is no consensus in the literature on how to obtain a true measure of party positions, I follow much of the existing literature on voters’ perception of parties’ policy positions and rely on the CMP/MARPOR data (Volkens et al., 2020). To allow a direct comparison between parties’ manifestos and coalition agreements, I apply the same coding scheme to coalition agreements that the widely used CMP/MARPOR project uses for parties. Specifically, the coalition agreement is first ‘unitised’ following the rules applied by the CMP/MARPOR to party manifestos so that the coalition agreement is also cut into a quasi-sentence. In a second step, a native speaker of the country of study checks, for each quasi-sentence, whether this is also included in one of the coalition partners’ manifestos. If the sentence is the exact same in the coalition agreement and the party manifestos, the coder simply assigns to the sentence contained in the coalition programme the same CMP/MARPOR coding assigned in the party manifestos; if the sentence is contained in one of the party manifestos but it is not exactly the same (for example it is longer or shorter), the coder carefully checks whether the meaning (=policy goal) of the sentence is the same or not. If the meaning is the same then the coder simply assigns to the sentence contained in the coalition programme the same CMP/MARPOR coding already assigned in the party manifesto. If the meaning is not the same then the coder independently assigns to the sentence 1 of the 56 standard categories of the CMP project.^8^

For the regression models presented in the paper, I rely on a widely employed method to measure left-right positions, namely the ‘RILE’-index – an index of *ri*ght-*le*ft positions of parties originally developed by Laver and Budge (1992). Table A1 in the Appendix shows the categories defined as left and right according to the RILE index. The formula to aggregate the scores of the 24 categories to a common score is to take the sum of the per-variables of all right-wing categories and subtract the sum of all left-wing categories. The CMP/MARPOR scores for the position of the party and the coalition respectively can potentially range from −100 to +100. In the analysis, I follow Adams et al. (2016) among others and recalibrate the CMP/MARPOR coding of party manifestos to match the scale of the election surveys.

The second key independent variable is constructed exactly as the first one but relying exclusively on voters’ perceptions. Hence, the second key independent variable is the *difference* between two differences: one between the ideological position of the voter ( $V_{before}$ ) and the perceived position of the coalition ( $C_{before}$ ) and the second between the ideological position of the voter ( $V_{before}$ ) and the perceived position of the party signing the agreement ( $P_{before}$ ), that is:


$$change\;in\;perceived\;distance=\left|V_{before}-C_{\left(perceived\right)before}\right|-\left|V_{before}-P_{\left(perceived\right)before}\right|$$

Since respondents in the surveys are asked to position parties using an 11-point scale from 0 = left to 10 = right, the ideological position of the party ( $P_{\left(perceived\right)before})$ is taken directly from the national election surveys used in this paper. For the perceived position of the coalition, I calculated, again for each respondent, the mean of the positions of the parties composing the coalition.^9^ Also for the perceived ideological position of the coalition, I rely on the pre-electoral measurement for two main reasons. First, this choice is dictated by the fact that respondents were not asked to position parties again in the post-election survey; second because a pre-election measurement allows us to minimise the possibility that voters’ perceptions of where a coalition stands are dependent on where the coalition actually stand after the election. The theoretical mechanism behind *Hypothesis 2* in fact expects voters to react exclusively on their perceptions.^10^

The third key independent variable is again a *difference* but this time in coalition preferences respectively before and after the election.


$$change\;in\;coalition\;preferences=\;CP_{after}-CP_{before}$$

Since election studies only measure coalition preferences directly during the pre-election wave, I employ the mean voter preference for the two parties forming the government to construct a measure of coalition preferences (respectively before and after the election).^11^ The intuition behind the use of this variable is the following: the change in party preferences before and after the elections might simply be due to a change in how much the respondents prefer a specific government to be formed. A positive change means that voters react positively to the formation of the government and this should have a positive effect on party preferences in line with *Hypothesis 3*.

Since the dependent variable measures changes at the individual level, I do not need to control for standard socio-demographic variables like age or gender that do not change at the individual level between the two survey waves. Adding control variables to the models does not alter the substantive conclusions discussed below. In the models, I add fixed effect by country-election to account for any heterogeneity in the data due to election-specific factors.

## Descriptive overview

**Figure 2.**
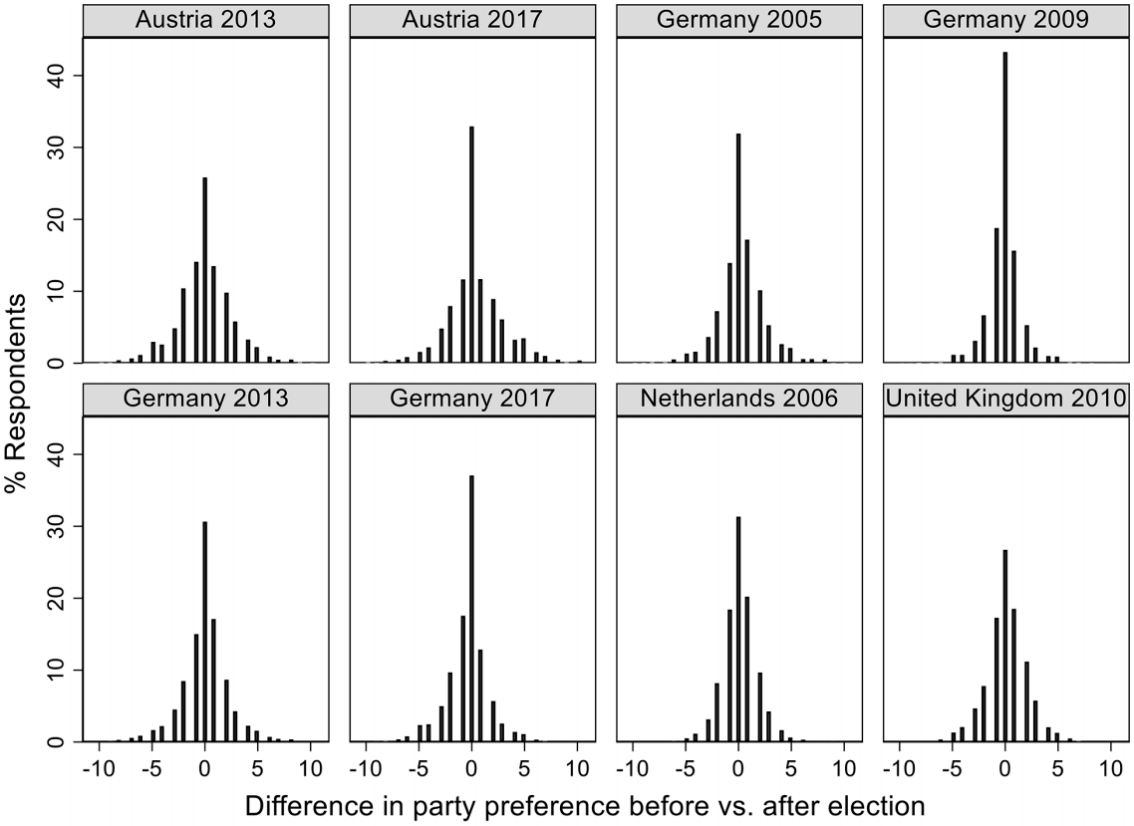
Distribution of the dependent variable. *Notes*: The figure displays the changes in party preferences after the elections.

**Figure 3.**
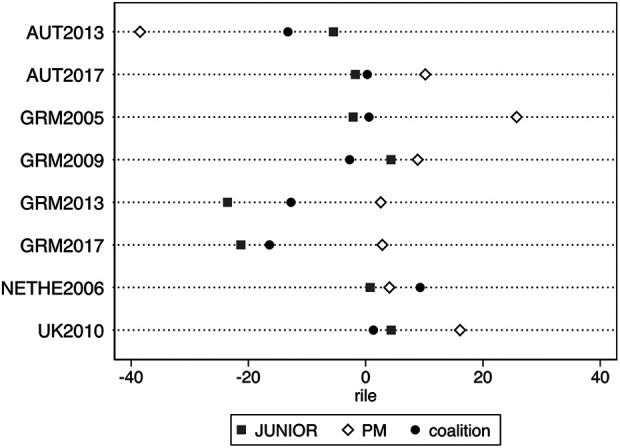
Positions of parties and coalitions in the countries examined. *Notes*: The figure displays the positions of the parties and the respective coalition for each election-year under investigation in this paper.

Figure 2 displays the distribution of the dependent variable. It shows a rather bell-shaped distribution centred around 0 with the majority of respondents between −5 and +5 intervals. About 30% of the respondents in each country do not change their party preferences at all after the elections; in Germany this percentage is a little higher, at 40%. More than half of the sample shows deviations from pre-election values almost equally on either the positive or the negative side of the distribution. A simple t-test reveals that the mean of post-electoral party preferences is statistically higher than the mean preference before the election for the prime minister’s party (*t = −5.1003, p < 0.000*) but not for the junior coalition partner and opposition parties. In the latter two cases, there is actually a decrease in party preferences after the election (*t =3.4700, p < 0.000* and *t = 4.4677, p < 0.000* respectively). This suggests that about half of the respondents do change party preferences, they like the largest, winning parties more than they did before the election; for the other half, there is instead a slight decrease in party preferences displays the positions of the parties and the respective coalition for each election-year under investigation in this paper. It shows how parties have moved compared to the pre-electoral stage when signing the coalition agreements. Figure 3 shows variation in terms of where the coalition stands vis-à-vis the coalition partners: in most cases the position of the coalition is slightly closer to the junior coalition partner. In the remaining two cases – Germany 2009 and the Netherlands 2006 – the position of the coalition is slightly closer to the prime minister’s party.

From Figure 3 it seems that the seat share is much more important in predicting the position of the coalition for more typical large party-small partner coalitions compared to grand-coalition governments. This makes sense since it is known that the prime minister’s party has far less control over its equally powerful partner under grand-coalition governments (e.g., Miller and Müller, 2010). In a more typical large party-small partner coalition, it seems that smaller coalition parties have disproportional influence on coalition policy in line with what voters seem to perceive in terms of coalition politics (Bowler et al., 2020). All in all, the results displayed in Figure 3 meet face validity and give confidence in the coding scheme used to code the coalition agreements.^12^

## Empirical findings

Turning to the multivariate analysis, Table 2 shows the effect of the three key independent variables on a change in party preferences, the dependent variable. Starting with the independent variable measuring actual change in the distance voter-party after the election. In both Model 1 (M1) and Model 5 (M5) in Table 2, the coefficient of the key independent variable measuring the distance voter-party before and after the election is negative, a result that largely supports *Hypothesis 1*: the more the parties move away from the voter compared to the distance before the election, the larger the decrease in party preferences after the election. The effect is statistically significant only for the prime minister’s party albeit the coefficient for the junior coalition partner is far larger. The total effect is rather small especially for the prime minister’s party: the coefficient in Model 1 tells us that there is a decrease in party preferences of about 0.06 points (on a scale from −10 to +10) for one unit decrease in the distance variable (that theoretically ranges from −10 to +10). This also means that to observe a conspicuous decrease in party preferences after a coalition agreement is signed, the prime minister’s party has to move considerably from its pre-electoral position to meet coalition partners’ requests.

**Table 2.** The effect of coalition agreements on party preferences: OLS models.

	**Prime Minister party**				**Junior partner**			
	(M1)	(M2)	(M3)	(M4)	(M5)	(M6)	(M7)	(M8)
Change actual distance	−0.056** (0.018)			0.012 (0.013)	−0.114 (0.062)			0.060 (0.041)
Change perceived distance		−0.103*** (0.013)		−0.114*** (0.009)		−0.097*** (0.013)		−0.114*** (0.010)
Change coalition preferences			0.962*** (0.009)	0.963*** (0.009)			1.038*** (0.009)	1.039*** (0.009)
Constant	−0.405*** (0.104)	−0.394*** (0.104)	−0.091 (0.063)	−0.104 (0.062)	−0.208* (0.102)	−0.261** (0.101)	0.091 (0.063)	0.036 (0.062)
Country Fixed-Effects	*Yes*	*Yes*	*Yes*	*Yes*	*Yes*	*Yes*	*Yes*	*Yes*
*N*	23486	23486	23486	23486	23486	23486	23486	23486
Adjusted R^2^	0.017	0.022	0.619	0.625	0.028	0.032	0.657	0.662
AIC	98182.9	98074.1	75950.7	75581.2	100437.1	100344.8	75950.7	75598.8

*Notes*: Standard errors in parentheses: * p < .05, ** p < .01, *** p < .001. The dependent variable is changes in party preferences after the elections.

Moving to the second key variable, Model 2 and Model 6 show that the more the coalition is perceived to be far away from the party, the larger the decrease in party preferences after the election. The effect is statistically significant both for the prime minister’s party and the junior coalition partner but slightly larger in the former case. These findings provide overall support for *Hypothesis 2.* The explained variance (as captured by the R-squared) is similar for both Models 1 and 5 and Models 2 and 6. When it comes to the third key independent variable, Model 3 and Model 7 indicate that a positive change of coalition preferences is related to a positive change in party preferences everything else being constant, hence supporting *Hypothesis 3*.^13^ Since all key independent variables have the same (theoretical) range of value, I can compare directly the effect of coalition preferences on the dependent variables vis-à-vis the effect of the ideological distance variables. It is clear from Table 2 that the effect of the variable measuring coalition preferences is much stronger than that of ideological distance and even more so for the junior coalition partner compared to the prime minister’s party. The full models (Model 4 and Model 8 respectively for the prime minister’s party and the junior coalition partner) further indicate that when controlling for both ideological distances – actual and perceived – the perceived ideological distance is the only one that matters.

The results point towards two main findings. First, in the formation of coalition governments, voters respond first and foremost to parties’ action itself and in terms of the preferences they have for the parties forming the government. Second, voters are not oblivious to the ideological shifts parties have to make to join a coalition government, which may ultimately be considered good news as it indicates that the mass-party ‘electoral connection’ extends beyond Election Day. However, the results also point to the conclusion that perceptions override actual distances, which casts a negative light on this paper’s findings since it is known that often perceptions may substantially diverge from actual distances.

## Extensions

In the analysis thus far, I have assumed a somewhat homogenous group of voters. There are, however, at least two grounds for further considering the reactions to coalition deals. First, the distinction between core supporters and non-core supporters may matter in terms of how much they care and hence might respond to the ideological movements of their ‘own’ parties. Since core supporters have the strongest ties to a party, its ideology and its issue positions, and given their strong identification with a party, they should be less likely to accept policy concessions than other party supporters. Figure A1 and Table A2 in the Appendix show that, when it comes to a change in distance, there is basically no difference between voters and non-voters for the prime minister’s party; on the other hand, voters of the junior coalition partners appear to be more lenient of their own party’s movements to form coalition governments, possibly suggesting that they might be more office-seeking than previously thought (e.g., Adams at al., 2006). Moving to ideology, voters and non-voters display no substantial difference. Finally, voters are systematically less likely to show a positive change in party preferences compared to non-voters after the elections holding all other variables constant at their mean. This is simply a matter of ceiling effect: voters have systematically much higher party and coalition preferences at the pre-electoral stage than non-voters so a positive change after the election is overall more difficult.

A second aspect concerns political knowledge. It is reasonable to expect that, all else being equal, voters are more likely to ‘react’ negatively to a change in the actual ideological distance when they know more. I operationalise political knowledge using survey questions asking respondents to place parties on a left-right scale. The measure is intuitively attractive since it concerns information necessary to understand and navigate successfully in the post-electoral theoretical mechanism put forwards by *Hypothesis 1*. Following Gordon and Segura (1997) and Munger et al. (2020) among others, this variable is generated for each of the pair-wise placements on this left-right scale. Since respondents are asked to place the main five parties in each country on the scale, this gives us 10 pair-wise comparisons for each country (except for the UK in which case respondents are asked to place only the three main parties). To decide which relative placement is correct, I use exogenous data collected by the Chapel Hill Research Group. Hence, respondents are given 1 point for a correct placement and 0 for all other placements. Then the number of correct placements is computed for each respondent. Figure A2 and Table A3 in the Appendix show that the negative effect of moving away from voters is felt more when political knowledge is high compared to when it is low, but the effect fails to reach the conventional level of statistical significance. There is basically no moderating effect of political knowledge for ideology nor changes of coalition preferences.

Finally, I have also tested for the possibility that *perceived* ideological distance might moderate the effect of the *actual* ideological distance between the voter and the party. The results presented in the Appendix, Figure A3 show that there is no moderation in the case of the prime minister party and a slightly significant moderation effect in the case of the junior coalition partner.

## Conclusion

Elections in multiparty democracies are most often followed by a discussion among parties that, despite having diverging ideological positions, must compromise over a common coalition agreement to form a government. Do voters respond to actual coalition deals and if so how?

In this paper, I focused first of all on the outcome of coalition talks in terms of the *actual* ideological movements that parties have to make after the election to sign a coalition deal. To this end, the position of the parties during the election campaign (as measured via their party manifestos) is compared to the position they hold after the election (as measured via the coalition manifesto). These two ideological positions are compared respectively before and after the election with the position of the voters. The analyses are performed combining existing panel survey data interviewing the same respondents before and after the same election with original content analysis of pre-electoral party manifestos and post-electoral coalition agreements. The broad expectation is that as parties move away from their voters, the lower utility that this implies for the voters will be reflected in a decrease in party preferences. Two alternative hypotheses state that voters do not respond to the actual ideological shifts parties make to join a coalition government but only to the action of government formation itself. Overall, this means that voters will react but in line with either their own perceptions of where parties stand once they have joined the government or even more simply in line with their preferences.

The results of this paper show that the more parties more away from voters after the elections when signing a coalition agreement, the more likely voters are to decrease their party preferences as they will derive a lower utility from voting for them. The results, however, further indicate that what matters primarily is the action of government formation per se. So there is a reaction in terms of ideological movements but perceptions of movements play a much more specific role than the actual movements derived from the analysis of parties’ and coalitions’ manifestos. Coalition preferences moderate voters’ reaction suggesting that the consequences of ideological shifts are somewhat weaker for those who like the parties and coalitions in the first place. Negative feelings for one of the compromising parties can make voters less likely to accept coalition compromises regardless of ideological positions. In this regard, the results are twofold in the sense that some parties may actually gain some support by getting closer to some of the voters that hitherto were far away from them before the elections. Further research should look into how much and perhaps in which direction parties can move before they get punished by (their) voters. Specific party characteristics – such as previous government experience or party nicheness – might mitigate how voters respond to their own party’s movement.

In terms of broader contribution, this paper highlights that voters do react to government agreements, but large shifts are required before voters will notice any real difference compared to what parties have promised during election campaigns. The paper opens up many interesting avenues of research. For instance, it is worth considering not only how much parties move away from previously held ideological positions but also how much they are successful in terms of election pledges or portfolio allocation (see Greene et al., 2020). And how lasting the effects of such coalition deals are.

Another dimension concerns the arguments that political parties use to ‘communicate’ and ‘justify’ compromises to voters. Such justificatory tactics might be especially important when parties cross ideological blocks (Falcó Gimeno and Fernandez-Vazquez, 2019). Some coalitions are much more surprising to voters than others and as such they should elicit a larger shift in preferences. Future studies should test for this possibility by taking into account a larger number of cases than done in this paper.

An increasingly high number of parties resort to a vote among their members before agreeing coalition deals: for example Germany’s Social Democrat party held a vote in 2018 asking its members whether to join Chancellor Angela Merkel’s grand coalition. The extent to which such a members’ vote might mitigate national voters’ response is surely an interesting aspect worth investigation.

All in all, I believe that this paper’s findings contribute to a better understanding of voters’ reactions to coalition government formation, an area of research that cries out for more study.

## Supplemental material

Supplemental Material, sj-pdf-1-ppq-10.1177_13540688211029794 - Voters’ short-term responsiveness to coalition deals Click here for additional data file. Supplemental Material, sj-pdf-1-ppq-10.1177_13540688211029794 for Voters’ short-term responsiveness to coalition deals by Carolina Plescia in Party Politics

## Notes

1. Minority governments also require compromise albeit in the form of external support (Strøm, 1990).

2. See Plescia et al. (2021) for a recent exception.

3. In this paper, I focus on the expected policy pay-offs during coalition talks (Debus and Müller, 2014). Voters may also focus on other bargaining outcomes such as portfolio allocation (see Greene et al., 2020). I discuss this additional possibility in the concluding section of this paper.

4. For instance, if 83 days have passed from Election Day to the end of coalition talks, all those interviewed the day after Election Day receive a weight equal to 1/83, those interviewed 2 days after will receive a weight equal to 2/83 and so on until the day coalition talks have ended with the signing of the agreement.

5. Substantive conclusions do not change if I do not use these weights at all.

6. There is no reason to include opposition parties since by definition distance variables do not vary in this case. For the Netherlands I only consider the largest junior coalition partner.

7. Five of the eight surveys included in this paper (Austria 2017, Germany in 2005, 2009 and 2013, and United Kingdom in 2010) have asked voters to position themselves on the left-right ideological scale not only in the pre-election wave but also after the election. The correlation between voter position before the elections and afterwards is relatively high *Pearson R* = 0.70 albeit differences exist across surveys.

8. Intercoder reliability run on a selection of 713 statements shows an agreement of 66% with a Kappa of 0.61 and associated standard error of 0.002.

9. Please note that using a weighted mean instead, where the weights are the cabinet seats proportional to legislative seats (Gamson, 1961) leads to identical conclusions.

10. The actual distance is positively correlated with the perceived distance albeit the correlation coefficient is quite low (*Pearson R* = 0.25 for the prime minister’s party and *Pearson R* = 0.29 for the junior partner).

11. The correlation between coalition preferences and the mean preference of the parties forming that coalition in the pre-electoral wave is usually above *Pearson R* = 0.70. While it is plausible that a change in party preferences may influence a change in coalition preferences, the strong correlation between coalition and party preferences actually means that, if anything, we are biasing our results against *Hypothesis 1*.

12. In three cases the position of the coalition is not between the party positions. In the Netherlands, this is due to the fact that while the coalition was formed by three parties (CDA, PvdA and Christian Union) I only consider the two largest coalition partners due to the few voters for the Christian Union in the survey. The position of the Christian Union that Figure 2 does not display is the farthest on the right and explains the position of the coalition slightly on the right compared to the two coalition partners I consider. For UK the position of the coalition is not substantially different from that of the junior coalition partner while for Germany 2009 I can only speculate that this is due to the fact that the CDU was unable to get rid of some of the positions the party had to take while ruling in a grand-coalition government with the SPD in the previous legislative term.

13. Note that using change in party preferences rather than coalition preferences to measure changes in coalition preferences leads to substantially similar findings (see Table A5 in the Appendix).
