# Supplementary material for: Voters’ short-term responsiveness to coalition deals
Source: Party Politics. 2021 Jul 5;28(5):927–38. doi: 10.1177/13540688211029794 (PMC9483677; doi:10.1177/13540688211029794)
Supplement: Supplemental Material, sj-pdf-1-ppq-10.1177_13540688211029794 - Voters’ short-term responsiveness to coalition deals [file sj-pdf-1-ppq-10.1177_13540688211029794.pdf]

ONLINE APPENDIX

Voters' short-term responsiveness to coalition deals

Table A1: Ideological positioning and CMP categories used

LEFT	RIGHT
103 Anti-Imperialism	104 Military: Positive
105 Military: Negative	201 Freedom and Human Rights
106 Peace	203 Constitutionalism: Positive
107 Internationalism: Positive	305 Political Authority
202 Democracy	401 Free Market Economy
403 Market Regulation	402 Incentives: Positive
404 Economic Planning	407 Protectionism: Negative
406 Protectionism: Positive	414 Economic Orthodoxy
412 Controlled Economy	505 Welfare State Limitation
413 Nationalisation	601 National Way of Life: Positive
504 Welfare State Expansion	603 Traditional Morality: Positive
506 Education Expansion	605 Law and Order: Positive
701 Labour Groups: Positive	606 Civic Mindedness: Positive

Figure A1: The average marginal effect of a change in preferences or distance for voters versus non-voters

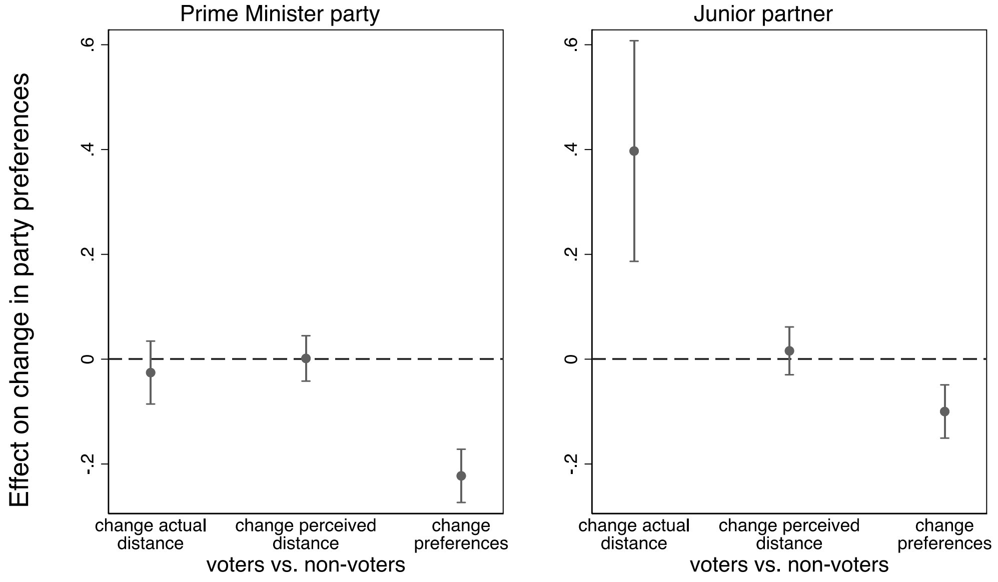

Notes: The dependent variable is changes in party preferences after the elections. Full results presented in Table A2.

Table A2: The effect of coalition agreements on party preferences for voters versus non-voters: OLS models

	**Prime Minister party**			**Junior partner**		
	(M1)	(M2)	(M3)	(M4)	(M5)	(M6)
Change actual distance	0.026	0.022	0.020	-0.001	0.072	0.060
	(0.014)	(0.013)	(0.013)	(0.044)	(0.041)	(0.041)
Change perceived distance	-0.107***	-0.107***	-0.105***	-0.095***	-0.100***	-0.094***
	(0.010)	(0.010)	(0.009)	(0.010)	(0.010)	(0.009)
Change coalition preference	0.962***	0.962***	0.996***	1.038***	1.038***	1.057***
	(0.009)	(0.009)	(0.010)	(0.009)	(0.009)	(0.009)
Voter=1	-0.164***	-0.169***	-0.214***	-0.300***	-0.301***	-0.315***
	(0.027)	(0.027)	(0.026)	(0.030)	(0.030)	(0.030)
Voter x Change actual distance	-0.026			0.397***		
	(0.031)			(0.107)		
Voter x Change perceived distance		0.002			0.016	
		(0.022)			(0.023)	
Voter x Change coalition preference			-0.223***			-0.100***
			(0.026)			(0.026)
Political knowledge	0.000	0.000	-0.001	0.002	0.003	0.003
	(0.006)	(0.006)	(0.006)	(0.006)	(0.006)	(0.006)
Country Fixed-Effects	*Yes*	*Yes*	*Yes*	*Yes*	*Yes*	*Yes*
Constant	-0.068	-0.066	-0.019	0.065	0.067	0.088
	(0.080)	(0.080)	(0.080)	(0.079)	(0.079)	(0.079)
N	23486	23486	23486	23486	23486	23486
Adjusted R2	0.626	0.626	0.630	0.665	0.665	0.666

Notes: Standard errors in parentheses: * p<.05, ** p<.01, *** p<.001. The dependent variable is changes in party preferences after the elections.

Figure A2: The average marginal effect of a change in preferences or distance for high versus low political knowledge

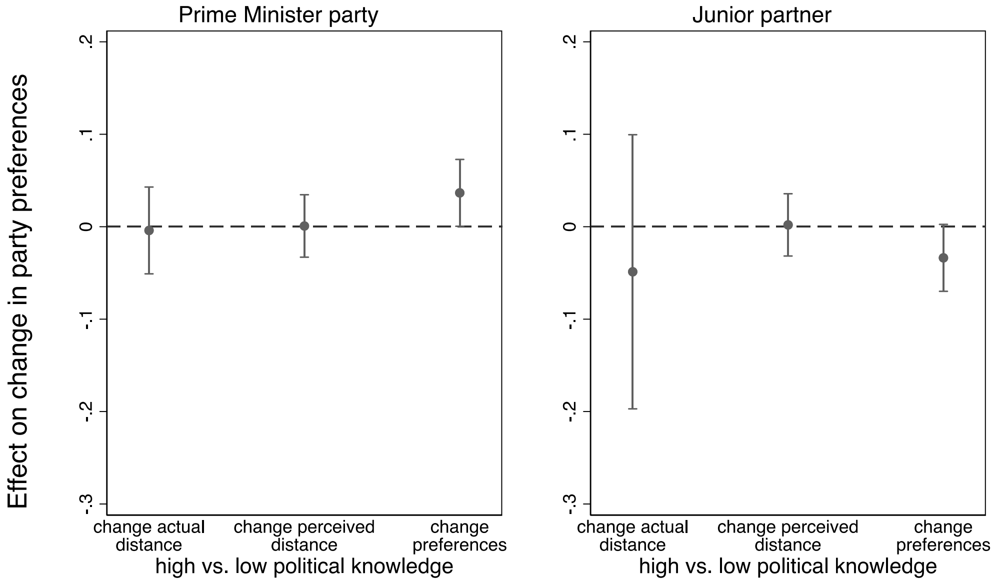

Notes: The dependent variable is changes in party preferences after the elections. Full results presented in Table A3.

Table A3: The effect of coalition agreements on party preferences mediated by political knowledge: OLS models

	**Prime Minister party**			**Junior partner**		
	(M1)	(M2)	(M3)	(M4)	(M5)	(M6)
Change actual distance	0.010	0.011	0.012	0.092	0.057	0.060
	(0.044)	(0.013)	(0.013)	(0.128)	(0.043)	(0.041)
Change perceived distance	-0.114***	-0.121***	-0.114***	-0.114***	-0.121***	-0.114***
	(0.009)	(0.030)	(0.009)	(0.010)	(0.030)	(0.010)
Change coalition preference	0.963***	0.963***	0.940***	1.039***	1.039***	1.059***
	(0.009)	(0.009)	(0.027)	(0.009)	(0.009)	(0.027)
Political knowledge	0.002	0.002	0.002	0.005	0.005	0.005
	(0.006)	(0.006)	(0.006)	(0.006)	(0.006)	(0.006)
Pol know x Change actual distance	0.000			-0.004		
	(0.005)			(0.015)		
Pol know x Change perceived distance		0.001			0.001	
		(0.004)			(0.004)	
Pol know x Change coalition preference			0.003			-0.002
			(0.003)			(0.003)
Country Fixed-Effects	*Yes*	*Yes*	*Yes*	*Yes*	*Yes*	*Yes*
Constant	-0.121	-0.123	-0.123	-0.005	-0.008	-0.004
	(0.079)	(0.079)	(0.079)	(0.079)	(0.079)	(0.079)
N	23486	23486	23486	23486	23486	23486
Adjusted R2	0.625	0.625	0.625	0.662	0.662	0.662

Notes: Standard errors in parentheses: * p<.05, ** p<.01, *** p<.001. The dependent variable is changes in party preferences after the elections.

Figure A3: The average marginal effect of change in actual distance for different levels of changes in perceived distance

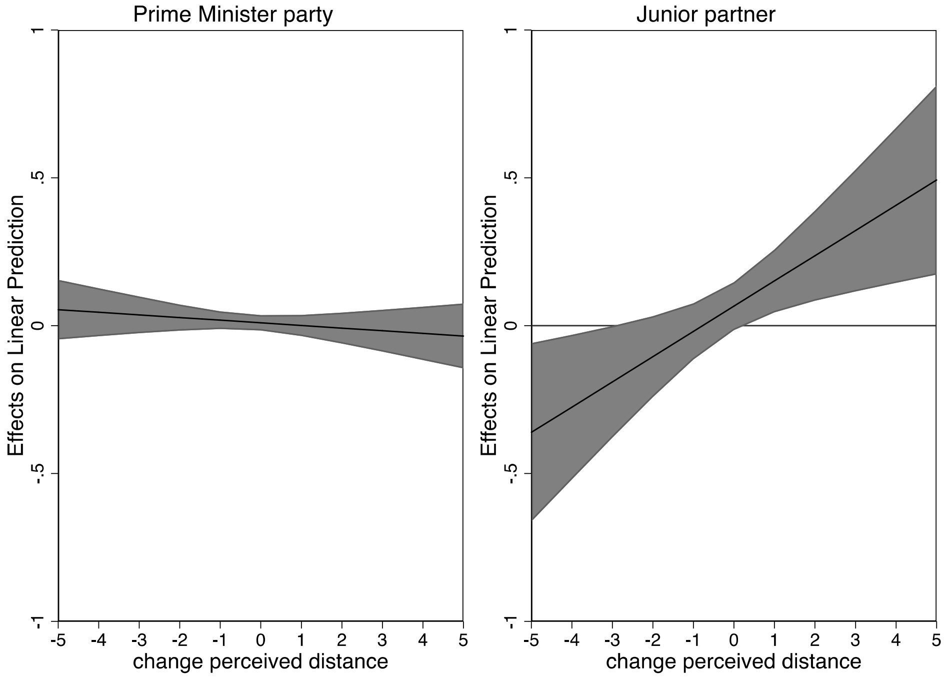

Notes: The dependent variable is changes in party preferences after the elections. Full results presented in Table A4.

Table A4: The effect of changes in perceived distance on party preferences mediated by changes in actual distance: OLS models

	**Prime Minister party**		**Junior partner**	
	(M1)	(M2)	(M3)	(M4)
Change actual distance	0.012	0.010	0.060	0.066
	(0.013)	(0.013)	(0.041)	(0.041)
Change perceived distance	-0.114***	-0.115***	-0.114***	-0.113***
	(0.009)	(0.009)	(0.010)	(0.010)
Change coalition preference	0.963***	0.963***	1.039***	1.039***
	(0.009)	(0.009)	(0.009)	(0.009)
Change actual distance X Change perceived distance		-0.009		0.085**
		(0.010)		(0.031)
Country Fixed-Effects	*Yes*	*Yes*	*Yes*	*Yes*
Constant	-0.104	-0.096	0.036	0.017
	(0.062)	(0.063)	(0.062)	(0.062)
N	23486	23486	23486	23486
Adjusted R2	0.625	0.625	0.662	0.662

Notes: Standard errors in parentheses: * p<.05, ** p<.01, *** p<.001. The dependent variable is changes in party preferences after the elections.

Table A5: Replication of Table 2 in the paper using change in party preferences rather than coalition preferences to measure changes in coalition preferences

	**Prime Minister party**		**Junior partner**	
	(M1)	(M2)	(M3)	(M4)
Change party preference Junior party	0.248***	0.255***		
	(0.010)	(0.010)		
Change party preference PM party			0.273***	0.280***
			(0.011)	(0.011)
Change actual distance		0.011		0.129
		(0.020)		(0.067)
Change perceived distance		-0.136***		-0.140***
		(0.014)		(0.015)
Country Fixed-Effects	*Yes*	*Yes*	*Yes*	*Yes*
Constant	-0.323**	-0.339***	-0.116	-0.185
	(0.100)	(0.100)	(0.098)	(0.097)
N	23486	23486	23486	23486
Adjusted R2	0.083	0.092	0.093	0.101

Notes: Standard errors in parentheses: * p<.05, ** p<.01, *** p<.001. The dependent variable is changes in party preferences after the elections.
